# RIG-I-like receptor-induced IRF3 mediated pathway of apoptosis (RIPA): a new antiviral pathway

**DOI:** 10.1007/s13238-016-0334-x

**Published:** 2016-11-04

**Authors:** Saurabh Chattopadhyay, Ganes C. Sen

**Affiliations:** 1 Department of Medical Microbiology and Immunology, University of Toledo College of Medicine and Life Sciences, 3000 Arlington Avenue, Mailstop 1021, Toledo, OH 43614 USA; 2 Cleveland Clinic, Department of Immunology, 9500 Euclid Avenue, NE20, Cleveland, OH 44195 USA

**Keywords:** RIPA, IRF3, innate immunity

## Abstract

The innate immune response is the first line of host defense to eliminate viral infection. Pattern recognition receptors in the cytosol, such as RIG-I-like receptors (RLR) and Nod-like receptors (NLR), and membrane bound Toll like receptors (TLR) detect viral infection and initiate transcription of a cohort of antiviral genes, including interferon (IFN) and interferon stimulated genes (ISGs), which ultimately block viral replication. Another mechanism to reduce viral spread is through RIPA, the RLR-induced IRF3-mediated pathway of apoptosis, which causes infected cells to undergo premature death. The transcription factor IRF3 can mediate cellular antiviral responses by both inducing antiviral genes and triggering apoptosis through the activation of RIPA. The mechanism of IRF3 activation in RIPA is distinct from that of transcriptional activation; it requires linear polyubiquitination of specific lysine residues of IRF3. Using RIPA-active, but transcriptionally inactive, IRF3 mutants, it was shown that RIPA can prevent viral replication and pathogenesis in mice.

## Introduction

We live in a world full of viruses and our immune system fights against them to keep us healthy and disease-free (Fensterl et al., 2015). The immune system is divided into two broad categories – the innate and the adaptive, both of which are required to protect against virus infection. The innate immune system, which is kicked off very early during virus infection, is the first line of antiviral defense; whereas the adaptive immune system, activated later, is dependent on the innate immune system. Therefore, an appropriate activation of the innate immune system is critical for the elimination of viruses, from an organism, by both branches of the immune system.

Type I interferon (IFN) mediates the key innate immune response against a wide range of viruses. The IFN system is triggered at the onset of virus infection via cellular recognition of viral components. Mammalian cells are equipped with protein sensors, e.g. TLRs, RLRs, NLRs or cGAS/STING, which detect the incoming virus particles in the cytoplasm or inside specific cellular compartments and trigger intracellular signaling pathways. These signaling pathways lead to the induction of IFN through the action of the transcription factor, Interferon Regulatory Factor 3 (IRF3). IFN is secreted from infected cells, so that it can inhibit virus replication in the infected, as well as neighboring uninfected, cells through the action of many antiviral proteins, the products of IFN-stimulated genes (ISG). ISGs can be directly induced by activated IRF3 as well, without any involvement of IFN. IRF3, a cytosolic protein, is inactive in uninfected cells; virus infection activates it by causing phosphorylation of its specific serine residues and its translocation to the nucleus, where it binds to the promoters of the target genes (Lin et al., 1998 and Sato et al., 1998). Specific cellular proteins, e.g. HDAC6, β-catenin and PKC-β, are required for full transcriptional activity of IRF3 (Chattopadhyay et al., 2013a).

**Figure 1:**
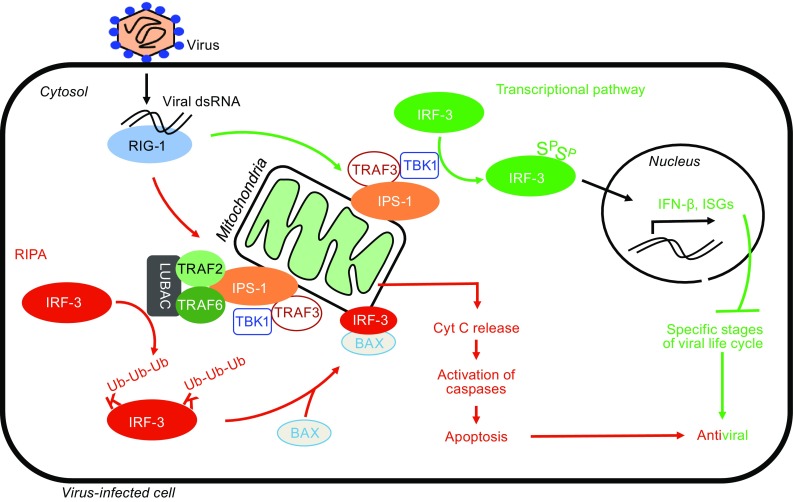
Dual functions of IRF3 in antiviral defense. Virus infection is recognized by the cytoplasmic sensor RIG-I, which binds to viral double-stranded RNA and triggers two signaling branches via mitochondrial adaptor IPS1. In the transcriptional pathway, IRF3 is translocated to the nucleus to induce antiviral genes, such as the interferon-beta (IFN-β) and interferon stimulated genes (ISGs). In contrast, in the RIPA branch, IRF3 is activated by LUBAC-mediated linear ubiquitination, which triggers its interaction with BAX to cause mitochondrial activation and apoptotic cell death. Both pathways contribute to the overall antiviral responses of the host

In addition to directly impairing virus replication, another effective protection mechanism used by multi-cellular organisms is to trigger suicide of the infected cell. Because only a few cells are initially infected, their premature death ensures that progeny viruses are too few to spread the infection efficiently. A critical discovery was made showing dual functions of IRF3 in virus infected cells: it not only induces antiviral genes but also triggers apoptotic cell death by the RLR-induced IRF3 mediated Pathway of Apoptosis (RIPA) (Fig. 1). Here, we discuss IRF3’s role in RIPA, the antiviral apoptotic pathway.

## Discovery of RIPA

It was observed that Sendai virus infected cells die by apoptosis and curiously, IRF3, but not IFN, is required for this response (Peters et al., 2008). The original thought was that IRF3, being a transcription factor, induces one or more pro-apoptotic proteins in the infected cell; but this idea was proved to be wrong when it was discovered that the transcriptional activity of IRF3 is not required for triggering apoptosis (Chattopadhyay and Sen, 2010b). However, like activation as a transcription factor, the apoptotic activity of IRF3 requires its activation by the RLR-signaling pathway; hence the name RIPA (Chattopadhyay et al., 2011). In RIPA, activated IRF3 binds, through the BH3 domain near its C-terminus, to the pro-apoptotic protein Bax. The IRF3-Bax complex translocates to mitochondria and triggers the release of cytochrome C to the cytoplasm; consequent activation of the caspases causes apoptosis. In virus-infected cells, the action of RIPA is temporally regulated (White et al., 2011). Although RIPA is activated early after infection, the anti-apoptotic protein XIAP blocks apoptosis by preventing caspase activation; later in infection, XIAP is degraded and the block to apoptosis is removed. Moreover, the stability of XIAP is regulated by PI3 kinase, which is activated by virus infection. The temporal regulation of RIPA indicates that viruses counteract it to prevent early death of the infected cell causing abortive virus replication. In the absence of RIPA, cells are persistently infected and they produce infectious progeny viruses, even in the presence of IFN and transcriptionally active IRF3 (Chattopadhyay et al., 2013b), thus demonstrating the physiological importance of RIPA.

## RIPA, a new pathway of IRF3 activation

The discovery of RIPA prompted the investigation of its need for the transcriptional activity of IRF3. It was observed that many transcriptionally inactive mutants of IRF3 could still trigger RIPA, indicating the existence of an apoptotic signaling pathway, in which a new activity of IRF3 was required (Chattopadhyay and Sen 2010a, Chattopadhyay et al., 2010b and Chattopadhyay et al., 2011). To characterize the new pathway, genetically modified human and mouse cells, which are defective in specific signaling proteins, were used. These experiments revealed that RIPA requires, in addition to several common proteins shared by IRF3’s transcriptional activation, a few RIPA-specific components, such as TRAF2 and TRAF6.

The obvious next question was how IRF3 is activated in RIPA. To address this, a series of IRF3 mutants was screened for transcriptional and apoptotic activities. Many mutants were active in one pathway, but not the other, demonstrating that the two activities of IRF3 are not inter-dependent. The screen led to the identification of specific lysine residues of IRF3 that are required for RIPA, but not the transcriptional pathway. Among 14 lysine residues, lysine 193 and lysine 313 or 315 of human IRF3 and lysine 188 and lysine 306 or 308 of mouse IRF3 are not only necessary, but sufficient for RIPA. The requirements of the specific lysines and additional TRAF proteins, some of which are ubiquitin E3 ligases, prompted an investigation of the need for ubiquitination of IRF3 in RIPA. Ubiquitination is a posttranslational modification by which specific lysine residues of the target protein are conjugated to polyubiquitin chains, using specific cellular ubiquitin conjugation systems. The fate of ubiquitinated proteins are dictated by the type of ubiquitin chains, which can be destructive and non-destructive. Detailed biochemical analyses revealed that a non-destructive linear polyubiquitination of two specific lysine residues (193, 313/315) of human IRF3 is sufficient to confer its RIPA activity; a minimal IRF3 mutant, harboring only those two specific lysine residues can activate RIPA. The required modification is catalyzed by the ubiquitinating complex, LUBAC; the RLR-activated IRF3 is recruited to LUBAC via TRAF2- and TRAF6-dependent mechanism. As expected, this newly identified modification of IRF3 is not needed for its transcriptional activity (Chattopadhyay et al., 2016).

## RIPA and viral pathogenesis

The discovery of a new function of IRF3 raised the question of whether RIPA can protect against viral replication and pathogenesis. To evaluate the antiviral activity of RIPA, the properties of those mutants of IRF3, which are active only in RIPA but not the transcriptional, pathway, were examined. Two such mutants of IRF3 exhibited antiviral activities against Sendai Virus (SeV) infection *in vitro*. These encouraging *in vitro* results prompted the engineering of a genetically modified (knock-in) mice, in which only RIPA, but not the transcriptional, branch of IRF3 is active. In these mice, antiviral genes are not induced, but the RIPA branch is functional. These mice were protected from respiratory dysfunction and resultant death caused by SeV infection (Chattopadhyay et al., 2016). We speculate that RIPA would protect against a variety of other viruses by triggering IRF3-dependent apoptotic cell death.

## Perspective

Our results indicate that both the transcriptional and the apoptotic activities of IRF3 contribute to its overall antiviral action, although the proportion of their contributions will vary among viruses and the infected cell types. It is difficult to assess the relative importance of the two antiviral branches in viral pathogenesis, *in vivo*; in genetically modified mice, the absence on one causes the other to compensate and appear to be more dominant than it really is in wild type mice. The new mouse model will be useful to specifically examine the impact of RIPA against other viral, bacterial, parasitic, and non-microbial, diseases. RIPA-like pathways of IRF3 have been reported in other disease models. A human retrovirus, HTLV1, triggers rapid apoptosis of primary monocytes using a RIPA-like pathway, which protects the cells from productive virus replication (Sze et al., 2013). Surprisingly, RIPA-like activity of IRF3 triggers apoptotic cell death of hepatocytes subjected to ethanol exposure, linking this activity to progression of alcoholic liver diseases (Petrasek et al., 2013). Future studies will reveal whether RIPA regulates other diseases, such as cancer, in which cellular apoptosis is a desired protective mechanism. It appears that, RIPA, which we discovered in the context of virus infection, is a potential regulator of non-viral pathogenesis as well.
